# Microarray and RNAi Analysis of P450s in *Anopheles gambiae* Male and Female Steroidogenic Tissues: *CYP307A1* Is Required for Ecdysteroid Synthesis

**DOI:** 10.1371/journal.pone.0079861

**Published:** 2013-12-04

**Authors:** Emilie Pondeville, Jean-Philippe David, Emilie Guittard, Annick Maria, Jean-Claude Jacques, Hilary Ranson, Catherine Bourgouin, Chantal Dauphin-Villemant

**Affiliations:** 1 Biogenèse des Stéroïdes, FRE2852, CNRS-UPMC, Paris, France; 2 Unit of Insect Vector Genetics and Genomics, Department of Parasitology and Mycology, CNRS Unit URA3012: Hosts, Vectors and Infectious Agents, Institut Pasteur, Paris, France; 3 Department of Vector Biology, Liverpool School of Tropical Medicine, Liverpool, United Kingdom; 4 Laboratoire d'Ecologie Alpine, UMR 5553, CNRS-Université de Grenoble, Grenoble, France; 5 Centre de Production et d'Infection des Anophèles, Institut Pasteur, Paris, France; 6 Department of Ecology and Evolution, Université de Lausanne, Lausanne, Suisse; New Mexico State University, United States of America

## Abstract

In insects, the steroid hormone 20-hydroxyecdysone (20E) coordinates major developmental transitions. While the first and the final steps of 20E biosynthesis are characterized, the pathway from 7-dehydrocholesterol to 5β-ketodiol, commonly referred as the “black box”, remains hypothetical and whether there are still unidentified enzymes is unknown. The black box would include some oxidative steps, which are believed to be mediated by P450 enzymes. To identify new enzyme(s) involved in steroid synthesis, we analyzed by small-scale microarray the expression of all the genes encoding P450 enzymes of the malaria mosquito *Anopheles gambiae* in active steroidogenic organs of adults, ovaries from blood-fed females and male reproductive tracts, compared to inactive steroidogenic organs, ovaries from non-blood-fed females. Some genes encoding P450 enzymes were specifically overexpressed in female ovaries after a blood-meal or in male reproductive tracts but only three genes were found to be overexpressed in active steroidogenic organs of both females and males: *cyp307a1*, *cyp4g16* and *cyp6n1*. Among these genes, only *cyp307a1* has an expression pattern similar to other mosquito steroidogenic genes. Moreover, loss-of-function by transient RNAi targeting *cyp307a1* disrupted ecdysteroid production demonstrating that this gene is required for ecdysteroid biosynthesis in *Anopheles gambiae*.

## Introduction

**Figure 1:**
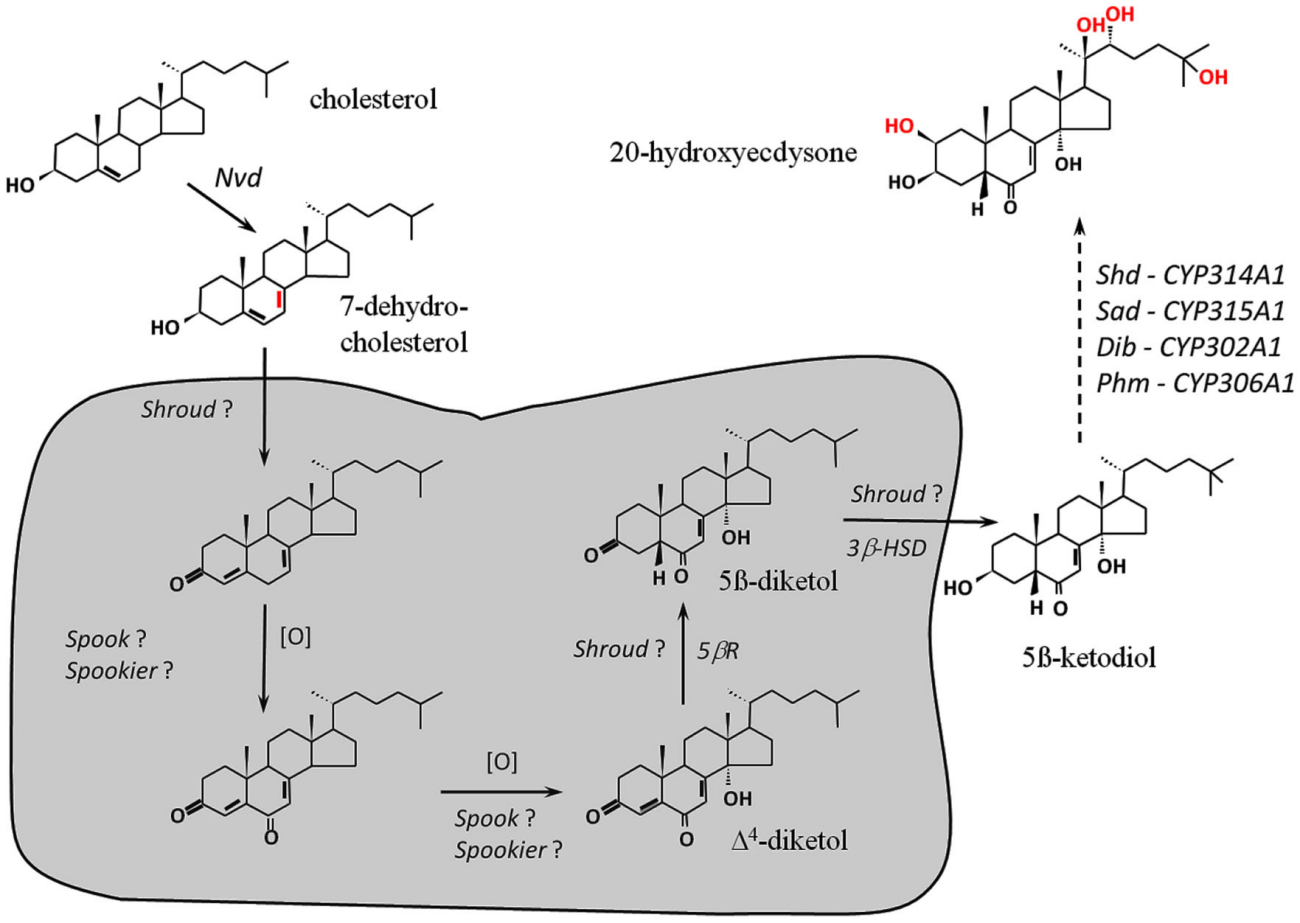
Biosynthetic pathway of ecdysteroids. From cholesterol to 20-hydroxyecdysone, the active steroid hormone. Characterized steps: *Nvd, neverland*, Rieske-domain oxygenase; *Phm, Phantom*, CYP306A1, 25-hydroxylase; *Dib, Disembodied*, CYP302A1, 22-hydroxylase; *Sad, Shadow*, CYP315A1, 2-hydroxylase; *Shd, Shade*, CYP314A1, 20-hydroxylase. Chemical modifications are shown in red on molecules. Putative steps of the “black box”, from 7-dehydro-cholesterol to 5ß-ketodiol, are represented within the dark grey box. [O] indicates oxidative step that might be catalyzed by a CYP enzyme. Some steps could be catalyzed by shroud, spook (CYP307A1) or spookier (CYP307A2). 5ßR: 5ß-reductase. 3ßHSD: 3ß-hydroxysteroid-dehydrogenase. Modified from Lafont *et al.* [4].

In insects and other arthropods, specific steroid hormones, called ecdysteroids, play a major role during growth, development and reproduction [1]–[5]. The prohormone ecdysone (E) is synthesized from dietary cholesterol (C) via a series of hydroxylation and oxidation steps in steroidogenic tissues, the prothoracic glands (PG) during post-embryonic development and the ovary of adults [4], [6]. E is further converted into the active hormone 20-hydroxyecdysone (20E) in target tissues. During the last decade, molecular genetic studies in *Drosophila melanogaster* have led to the identification and characterization of several genes involved in 20E biosynthesis (Figure 1). The first enzymatic step, *i.e.* the conversion of C into 7-dehydrocholesterol (7dC), is catalyzed by the Rieske-domain oxygenase Neverland (Nvd) [7]–[10]. The last four hydroxylation steps, from 5β-ketodiol to 20E, are catalyzed by four P450 enzymes (CYPs): CYP306A1 (Phantom; Phm) [11]–[12], CYP302A1 (Disembodied; Dib) [13]–[14], CYP315A1 (Shadow; Sad) [14] and CYP314A1 (Shade; Shd) [15]. The genes encoding these four P450 enzymes were identified from study of *Drosophila* embryonic lethal mutants, the Halloween mutants, which exhibit ecdysteroid deficiency [13], [16].

While the above-mentioned steps of biosynthesis are well characterized, little is known about the conversion from 7dC to 5β-ketodiol, commonly referred as the “black box”, for which no stable intermediate has been identified. The hypothetic metabolic steps occurring in the black box imply modifications at multiple carbon positions (Figure 1, shaded part). This includes the oxidation of 3beta-alcohol to ketone, the oxidation of carbon 6 with concomitant loss of the 4beta- and 6-hydrogens to form the 6-keto group, and 14alpha-hydroxylation. Δ^4^-diketol would then be converted by a 5ß-reductase to 5ß-diketol further transformed in 5ß-ketodiol by a 3ß-reductase [4], [17]. The black box, and more particularly the oxidative steps, is thought to involve one or more P450 enzymes that still remain uncharacterized [6], [18]. Consistent with this hypothesis, CYP307A1 (Spook, Spo) and CYP307A2 (Spookier, Spok) have been proposed to catalyze one of the ecdysteroid biosynthesis oxidative steps [18]–[21]. The gene encoding CYP307A1 has been first described in the *Drosophila* Halloween mutants [13] and further identified in a differential display PCR screen in the PG of the Lepidoptera *Bombyx mori* [18]. In *D. melanogaster*, unlike other Halloween genes, *cyp307a1* is expressed only in embryos and in the follicle cells of ovary but not during the larval stages. However, its paralog *cyp307a2* is expressed within the PG cells during larval stages only and RNAi mediated reduction of its expression leads to developmental arrest at the first larval stage [19]. Ketotriol and ketodiol can rescue *cyp307a1* mutant embryos and *cyp307a2* knockdown larvae respectively, while C or 7dC do not, suggesting that *cyp307a1* and *cyp307a2* are likely to be components of the black box [19]. Recently, Niwa *et al.* [22] identified the *non-molting glossy* (*nm-g*)/*shroud* (*sro*) gene in *B. mori* and *D. melanogaster*, respectively. This gene encodes a short-chain dehydrogenase/reductase that seems to be also involved in the black box, as the application of ketodiol, but not C or 7dC, overcomes the larval arrest observed in *nm-g*/*sro* mutant animals. Similarly, *cyp6t3* constitutes another candidate gene in the black box as its knockdown in *Drosophila* PG leads to E deficiency phenotypes that can be rescued by feeding larvae with E or one of several E biosynthetic precursors [23]. However, *cyp6t3* has no clearly identifiable ortholog in other insect species, which is unusual compared to the characterized Halloween genes [23]–[24]. Even if these experiments tentatively place *cyp307a1* and *cyp307a2*, along with *cyp6t3* and *Sro*, inside the black box pathway, no specific enzymatic activity has been assigned yet to the corresponding proteins. Therefore, whether it remains unidentified enzymes responsible for ecdysteroid biosynthesis is still unknown.

In female mosquitoes, a blood meal triggers the ovaries to secrete high amounts of E, subsequently hydroxylated to 20E, which in turn activates the transcription of the vitellogenin (Vg) gene in the female fat body. This leads to the production and secretion of Vg proteins into the hemolymph, that are later incorporated into the growing oocytes [2], [25]. Among mosquitoes and more generally among insects, the malaria vector *Anopheles gambiae* appears so far unique because not only blood-fed (BF) females, but also males produce high amounts of 20E. In males, the steroid hormone is produced by and stored in the accessory glands (MAGs) to be further transferred to females during mating [26]. In both females and males, expression of the genes involved in the last steps of steroidogenesis is tightly correlated with ecdysteroid production [26] as described in several insect species [11]–[12], [27]–[31], as well as in crustaceans [32]. Taken together, all these results suggest that the timing of hormone production highly depends on transcriptional regulation of the enzymes involved in its biosynthesis. Due to the high steroidogenic capacities of *A. gambiae* females and males, this mosquito species then constitutes a good model to identify new genes involved in ecdysteroid biosynthesis. To uncover unidentified CYP(s) gene(s) involved in 20E biosynthesis in *A. gambiae*, we took advantage of a small-scale microarray, which was initially developed to study metabolic-based insecticide resistance in this malaria vector [33]. The microarray covers 230 genes of *A. gambiae*, including all members of the three main enzyme families involved in insecticide metabolism: the cytochrome P450 monooxygenases (CYPs), the gluthatione-S-transferases (GSTs) and the carboxylesterases (COEs). By comparing expression of *A. gambiae* CYP genes between a non active steroidogenic tissue, the ovaries from non blood-fed (NBF) females, and active steroidogenic tissues, ovaries from BF females or male reproductive tracts (MRTs), we identified 3 CYP genes significantly over-transcribed in both female and male steroidogenic tissues: *cyp4g16*, *cyp6n1* and *cyp307a1*. We demonstrate that only *cyp307a1* has the same expression pattern as other genes involved in steroid synthesis in *A. gambiae* [26]. Moreover, transient RNAi targeting *cyp307a1* significantly decreases E production in *A. gambiae* females. Overall, our results demonstrate that *cyp307a1* is required for ecdysteroid biosynthesis in *A. gambiae*.

## Results

### Expression of steroidogenic genes is increased in steroidogenic active *versus* steroidogenic inactive tissues

To identify new CYP(s) involved in ecdysteroid synthesis, changes in *A. gambiae* CYP transcription levels in gonads associated with steroidogenesis were assessed using the “*Anopheles* detox chip microarray” which contains probes for the major *A. gambiae* detoxification genes [33]. Because steroidogenic CYPs genes are usually up-regulated in active steroidogenic tissues [11]–[12], [26]–[32], we compared gene expression between ovaries of NBF females, which do not produce ecdysteroids, and ovaries at different times after the blood meal (5, 16, 22 h PBM) or MRTs, tissues which actively produce ecdysteroids [26]. Gene expression results obtained for the four steroidogenic *cyp*s previously identified, *i.e. cyp306a1*, *cyp302a1*, *cyp315a1* and *cyp314a1*, are given in Table 1. In ovaries, the transcription of *cyp306a1* and *cyp302a1* is significantly increased at 16 h and 22 h PBM. By contrast, *cyp315a1* and *cyp314a1*, involved in the last two steps of ecdysteroid biosynthesis, are downregulated at 16 h and 22 h PBM when ecdysteroid production peaks. In MRTs, *cyp306a1*, *cyp302a1* and *cyp314a1* are strongly overexpressed compared to ovaries of NBF females while *cyp315a1* is not significantly differently transcribed between MRTs and NBF ovaries. Overall, the earlier the steroidogenic genes are in the 20E biosynthetic pathway, the more they are up-regulated in steroidogenic active tissues. These results are in agreement with previous RT-PCR results [26] and validate the use of this microarray to identify genes encoding the early steps of steroidogenesis from the so-called “black box”.

**Table 1:** Expression of genes encoding steroidogenic CYP in steroidogenic ovaries and MRTs compared to non steroidogenic ovaries.

*Steroidogenic*	Ovaries	Ovaries	Ovaries	MRTs
*gene*	5 h PBM	16 h PBM	22 h PBM	
*CYP306A1*	0.98 - *9.45E-01*	**1.55 - ** ***1.24E-02***	**2.05 - ** ***1.29E-02***	**18.77 - ** ***3.82E-08***
*CYP302A1*	1.01 - *9.04E-01*	1.11 - *2.04E-01*	**1.29 - ** ***3.54E-02***	**5.44 - ** ***1.27E-02***
*CYP315A1*	0.93 - *2.57E-01*	0.79 - *4.88E-02*	0.67 - *2.40E-02*	0.86 - *0.65E-01*
*CYP314A1*	0.98 - *9.46E-01*	0.97 - *9.88E-01*	0.86 - *7.96E-01*	**2.86 - ** ***4.21E-05***

Expression ratios and p values (italic) for the genes encoding CYPs previously characterized as steroidogenic CYPs, in ovaries of blood-fed females at 5 h, 16 h, 22 h post blood-meal (PBM) and in male reproductive tracts (MRTs) compared to ovaries from non blood-fed females. CYP genes are listed according to their position in the 20E biosynthesis pathway. Genes showing a significant over transcription (ratio >1.5 and P<0.05) are shown in bold.

### *Cyp4g16*, *cyp6n1* and *cyp307a1* are significantly up-regulated in steroidogenic tissues

To identify candidate genes that could be involved in steroidogenesis, any *CYP* satisfying all of the following criteria was selected: (*i*) the gene is up-regulated with both a transcription ratio >1.5-fold and P value<0.05, (*ii*) the gene is up-regulated both in ovaries after a blood meal at any time point and in MRTs compared to ovaries of NBF females. Selecting only *CYPs* up-regulated in both steroidogenic ovaries and MRTs removed genes that may be involved in sex-specific gonad functions. Moreover, as steroidogenic CYPs are well conserved in insects while detoxification CYPs are not, using results obtained with two different strains (*Yaoundé* and *Kisumu*) of *A. gambiae* appears to be a good criteria for identifying steroidogenic CYPs. Such candidates are expected to be regulated in the same way in two different strains contrary to detoxification enzyme which might be differently regulated between two strains [34]–[35]. Of 103 P450 genes represented on the microarray, only 3 candidate CYPs met our screening criteria: *cyp4g16*, *cyp6n1* and *cyp307a1* (Table 2).

**Table 2:** CYP genes over transcribed in steroidogenic ovaries and/or in steroidogenic MRTs compared to non steroidogenic ovaries.

*Up-regulated genes*	Ovaries	Ovaries	Ovaries	MRTs
	5 h PBM	16 h PBM	22 h PBM	
*CYP12F1*	1.52 - *1.74E-01*	1.15 - *6.30E-01*	0.85 - *7.61E-01*	**1.92 - ** ***2.09E-02***
*CYP12F2*	0.76 - *1.25E-02*	0.73 - *7.25E-02*	0.61 - *2.78E-02*	**2.85 - ** ***6.41E-07***
*CYP12F3*	ND	ND	ND	**14.96 - ** ***1.87E-05***
*CYP12F4*	0.83 - *1.90E-01*	0.91 - *7.36E-01*	0.76 - *2.00E-01*	**16.67 - ** ***6.20E-04***
*CYP302A1*	1.01 - *9.04E-01*	1.11 - *2.04E-01*	1.29 - *3.54E-02*	**5.44 - ** ***1.27E-02***
*CYP305A2*	**1.50 - ** ***6.20E-03***	1.15 - *2.98E-01*	1.00 - *9.99E-01*	ND
*CYP306A1*	0.98 - *9.45E-01*	**1.55 - ** ***1.24E-02***	**2.05 - ** ***1.29E-02***	**18.77 - ** ***3.82E-08***
***CYP307A1***	1.32 - *7.77E-02*	1.28 - *1.01E-01*	**1.51 - ** ***4.41E-02***	**11.61 - ** ***9.30E-10***
*CYP314A1*	0.98 - *9.46E-01*	0.97 - *9.88E-01*	0.86 - *7.96E-01*	**2.86 - ** ***4.21E-05***
*CYP4AR1*	0.96 - *9.46E-01*	1.07 - *7.90E-01*	1.11 - *8.76E-01*	**1.95 - ** ***1.83E-02***
*CYP4D15*	ND	1.31 - *6.19E-02*	1.27 - *5.58E-01*	**2.14 - ** ***4.05E-03***
*CYP4D22*	0.89 - *8.690E-01*	0.99 - *9.99E-01*	ND	**8.46 - ** ***3.89E-08***
***CYP4G16***	**2.00 - ** ***<2.00E-16***	**2.32 - ** ***6.30E-03***	**2.22 - ** ***2.00E-04***	**3.31 - ** ***2.16E-02***
*CYP4J5*	0.93 - *7.50E-01*	0.90 - *8.76E-01*	0.84 - *7.61E-01*	**3.38 - ** ***5.97E-03***
*CYP4K2*	0.82 - *1.95E-01*	0.87 - *4.97E-01*	0.86 *- 7.61E-01*	**2.28 - ** ***2.00E-04***
*CYP6AF1/2*	**1.70 - ** ***7.70E-03***	1.02 - *9.93E-01*	0.97 - *9.83E-01*	ND
*CYP6AG1*	**2.33 - ** ***4.10E-03***	**1.82 - ** ***6.30E-03***	0.62 - *7.96E-01*	ND
*CYP6M2*	1.09 - *2.93E-01*	0.87 - *2.22E-01*	0.83 - *2.57E-01*	**6.95 - ** ***2.14E-05***
*CYP6M3*	1.22 - *1.44E-02*	0.82 - *1.19E-01*	0.67 - *1.29E-02*	**1.61 - ** ***1.36E-02***
*CYP6M4*	1.25 - *1.63E-01*	1.43 - *1.79E-01*	1.17 - *7.77E-01*	**6.61 - ** ***1.60E-04***
***CYP6N1***	0.91 - *4.15E-01*	**1.71 - ** ***<2.00E-16***	0.52 - *1.29E-02*	**5.99 - ** ***2.70E-04***
*CYP6P3*	0.92 - *6.19E-01*	1.23 - *8.28E-01*	0.89 - *8.10E-01*	**2.03 - ** ***7.58E-03***
*CYP6S1*	0.76 - *6.40E-03*	0.78 - *4.88E-02*	0.91 - *5.76E-01*	**7.43 - ** ***2.91E-07***
*CYP6S2*	0.91 - *2.930E-01*	1.04 - *8.93E-01*	1.19 - *1.72E-01*	**8.66 - ** ***1.54E-09***
*CYP6Z1*	ND	ND	ND	**3.04 - ** ***6.20E-04***
*CYP6Z2*	1.30 - *9.08E-02*	1.08 - *8.72E01*	0.85 - *7.96E-01*	**12.67 - ** ***2.25E-05***
*CYP9J5*	0.87 - *2.01E-01*	0.75 - *1.79E-01*	0.65 - *1.57E-01*	**2.52 - ** ***1.88E-03***
*CYP9K1*	0.92 - *2.95E-01*	0.90 - *4.81E-01*	0.92 - *7.61E-01*	**2.25 - ** ***6.85E-03***

Only CYP genes overexpressed in ovaries of blood-fed females at least in one time point and/or in MRTs compared to ovaries from NBF females are listed. Values in bold indicate a ratio >1.5 and a p value<0.05. ND: Not detected or detected in less than 2 arrays. CYP genes previously characterized as involved in steroidogenesis are underlined. Candidate genes, overexpressed in ovaries of BF females, at least in one time point, and in MRTs compared to ovaries from NBF females, are in bold. PBM: post-blood meal. MRTs: Male reproductive tracts.

### Only *cyp307a1* shows a typical steroidogenic enzyme expression pattern

**Figure 2:**
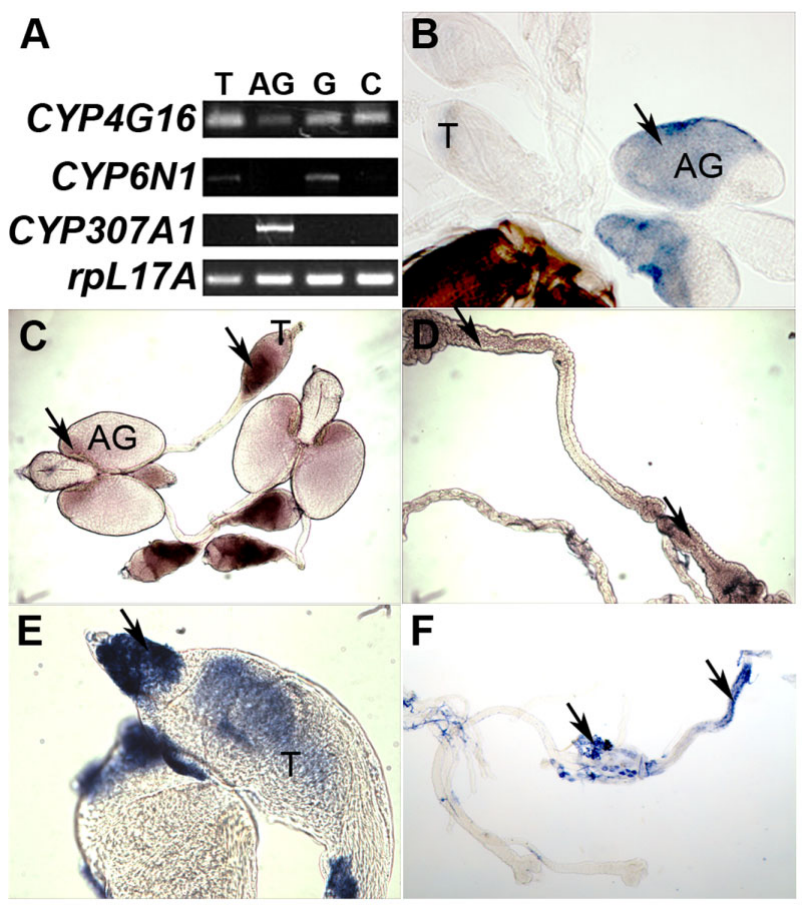
Expression pattern of *cyp4g16*, *cyp6n1* and *cyp307a1* in adult males. (A) RT-PCR analysis of *cyp4g16, cyp6n1* and *cyp307a1* expression pattern in males (T, testes; AG, accessory glands; G, gut and Malpighian tubules; C, carcass). *rpL17A* is used as a control gene. (B to F) *In situ* expression pattern of *cyp307a1*, *cyp4g16*, *cyp6n1* in males (T, testes; AG, accessory glands). (B) *cyp307a1* is detected in the anterior part of accessory glands. (C) *cyp4g16* is detected at the bottom of testes and in the posterior part of accessory glands. (D) *cyp4g16* is detected in the anterior and posterior gut. (E) *cyp6n1* is detected at the top of testes. (F) *cyp6n1* is expressed in the posterior gut and malpighian tubules. Black arrowheads show expression zones.

Expression of the three candidate genes was further analyzed by RT-PCR and *in situ* hybridization in different tissues of adult males (Figure 2) as steroidogenic enzyme gene expression is strictly restricted to the anterior part of MAGs in *Anopheles* males contrary to a broader expression in female tissues [26]. As shown in Figure 2A, *cyp4g16* is expressed in testes, MAGs, gut and carcass. *In situ* hybridization revealed that *cyp4g16* is expressed in the posterior part of the testicular follicular sheath and in the posterior part of the MAGs (Figure 2C), as well as in the anterior and posterior midgut (Figure 2D). *Cyp6n1* is mainly expressed in the testes and in the gut (Figure 2A, 2E, 2F). In contrast with *cyp4g16*, *cyp6n1* is expressed in the spermatogonies during early stages of spermatogenesis (Figure 2E). Unlike the two other candidate genes, expression of *cyp307a1* was restricted to the MAGs and more precisely to the anterior part of the glands (Figure 2B), as observed for other steroidogenic genes [26]. In conclusion, only *cyp307a1* shows a typical steroidogenic CYP expression pattern in *Anopheles* adult male and therefore appeared to be the most relevant candidate for functional validation.

### CYP307A1 is required for ecdysteroid production in *Anopheles gambiae*

**Figure 3:**
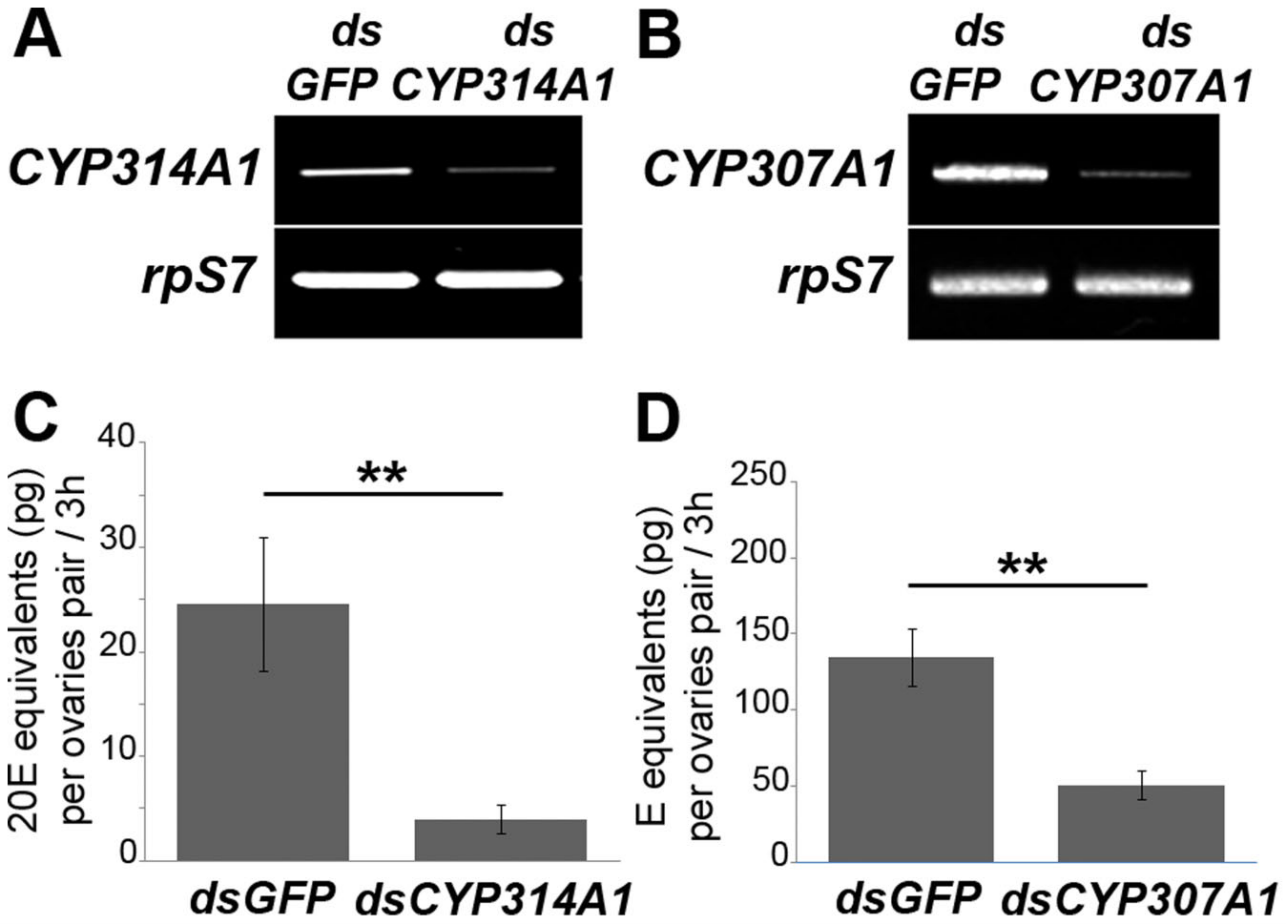
*In vitro* ecdysteroid secretion by ovaries of dsRNA injected females. (A) RT-PCR analysis of *cyp314aA1* in ovaries of ds*gfp* and ds*cyp314a1* females 22h after blood-feeding. (B) RT-PCR analysis of *cyp307a1* in ovaries of ds*gfp* and ds*cyp307aA1* females 22h after blood-feeding. (C) *In vitro* ecdysteroid secretion of ovaries from ds*gfp* and ds*cyp314a1* females 22 h after blood-feeding. Results are expressed as mean ± SEM in 20E equivalents (in pg) per ovaries pair. (D) *In vitro* ecdysteroid secretion of ovaries from ds*gfp* and ds*cyp307a1* females 22 h after blood-feeding. Results are expressed as mean ± SEM in E equivalents (in pg) per ovaries pair. Results were subjected to statistical analysis using Mann-Whitney test (**, *P*<0.01).

If CYP307A1 is indeed required for ecdysteroid biosynthesis in *A. gambiae*, knocking down *cyp307a1* expression should decrease ecdysteroid production by steroidogenic tissues. To test this hypothesis, we performed transient RNAi on *Anopheles* females targeting *cyp307a1* before measuring *in vitro* ovarian 20E production 22 h after blood-feeding. As a positive control, we first determined whether knocking-down by transient RNAi a known steroidogenic gene, *cyp314a1*, would indeed decrease 20E production in ovaries of BF females. As shown in Figure 3A, expression of *cyp314a1* was strongly decreased in ovaries from BF females injected with ds-*cyp314a1* compared to controls (ds-*gfp*-injected BF females). The decrease in *cyp314a1* RNA led to a significant reduction of ovarian 20E production in ds-*cyp314a1*-injected BF females compared to controls (Figure 3C). Therefore, transient RNAi targeting a steroidogenic enzyme gene in mosquito female is a powerful method to characterize steroidogenic genes. Injection of ds-*cyp307a1* also strongly decreased *cyp307a1* expression in ovaries from ds-*cyp307a1*-injected BF females compared to controls (ds-*gfp*-injected BF females) (Figure 3B). As depicted in Figure 3D, ovarian ecdysteroid production of ds-*cyp307a1*-injected females was also significantly decreased compared to controls, demonstrating that *cyp307a1* is required for ecdysone biosynthesis in *Anopheles*.

## Discussion

Our microarray analysis revealed a large set of genes encoding CYP overexpressed in ovaries of BF female and in male reproductive tracts. Among these genes and except the CYPs previously known to be involved in 20E biosynthesis, we identified 3 genes encoding P450 enzymes, *cyp4g16*, *cyp6n1* and *cyp307a1*, that are overexpressed in adult active steroidogenic tissues of both sexes, *i.e.* reproductive tracts of mature males and ovaries of BF females, compared to non active steroidogenic tissues. In addition, we demonstrated that, among these three genes, only *cyp307a1* has a similar expression pattern as other CYP genes involved in ecdysteroid biosynthesis in *A. gambiae* adults [26]. We further demonstrated that transient loss-of-function of *cyp307a1* leads to a decreased E production in *A. gambiae*, validating the involvement of *cyp307a1* in steroidogenesis in this mosquito species.

We found that the previously characterized genes *cyp306a1*, encoding the 25-hydroxylase, and *cyp302a1*, encoding the 22-hydroxylase, are up-regulated in ovaries of BF females from 16 h to 22 h PBM, time at which ovaries produce high amounts of steroids compared to ovaries from NBF females. In contrast, *cyp315a1* and *cyp314a1*, which encode respectively the 2- and the 20-hydroxylase, the two final steps leading to the active hormone 20E, are not significantly up-regulated in active steroidogenic ovaries *versus* non active ones. This is consistent with the fact that these genes are already expressed in ovaries of NBF females and also expressed in some peripheral tissues in *A. gambiae* and *D. melanogaster* [4], [14], [15], [26], [36]. The observation that these two final steps 20E biosynthesis, and not only the 20-hydroxylase, are not restricted to steroidogenic tissues compared to the earlier steps could possibly be correlated to the less polar nature of the final steroid compounds. Indeed, 2-deoxyecdysone (2dE) and E are more soluble compounds than earlier intermediates and are likely to easily diffuse from steroidogenic cells to target cells/tissues that would possess the capacity of converting 2dE into the biologically active 20E hormone. A similar situation has also been reported in crustaceans [37]. In MRTs, the strong overexpression of the genes involved in steroidogenesis, except *cyp315a1*, matches with the huge steroidogenic capacity of the accessory glands of *A. gambiae* males that exceeds by far that of vitellogenic ovaries [26]. As observed in active steroidogenic ovaries, *cyp315a1* is not overexpressed in steroidogenic MRTs. In contrast, MRTs overexpress *cyp314a1* (encoding the 20-hydroxylase). While in BF females, ovaries produce a mixture of E and 20E, MAGs, the steroidogenic tissue of MRT, produce the active hormone 20E. MAGs then represent a target tissue-like, which possess a strong 20-hydroxylase activity to ensure the production of large amounts of 20E that is then transferred to female during copulation [26].

With the exception of the CYPs previously known to be involved in 20E biosynthesis, only 3 additional CYP genes were found to be overexpressed in steroidogenic tissues of both females and males compared to non steroidogenic tissues: *cyp4g16*, *cyp6n1* and *cyp307a1*. For two main reasons, only *cyp307a1* was further investigated as a candidate gene in the steroid biosynthesis pathway in *A. gambiae* mosquitoes. First, we show here that only *cyp307a1* has the same expression pattern as the previous characterized genes being specifically expressed in the anterior part of the MAGs, the unique steroidogenic tissue in *A. gambiae* males [26]. In contrast, *cyp4g16* and *cyp6n1* are expressed mainly in the testes and in the gut, tissues that do not produce steroids. Secondly, the critical physiological function of steroidogenic enzymes has imposed constraints on their selection. As a consequence, *cyp* genes involved in steroid biosynthesis are well conserved among ecdysteroid producing animals [4], [20], [34]. In contrast, *CYP* genes involved in detoxification processes present a higher diversification among species, likely due to differences in their ecological niches and adaptive strategies [38]–[39]. Indeed, phylogenetic analyses showed that *cyp307a1* possesses a true ortholog in insect genomes, even if, in contrast with genes encoding the terminal hydroxylases which have a single ortholog in any arthropod species investigated so far, *cyp307a1* has also two paralogs, *cyp307a2* and *cyp307b1* [4], [20], [40]. Conversely, *cyp4g16* and *cyp6n1* do not show such a high degree of conservation in other insect species, suggesting that these two genes are not involved in a highly conserved metabolic pathway like steroid biosynthesis. As *cyp4g16* has been associated with insecticide-spraying periods in *Anopheles arabiensis* (*A. gambiae* complex) in Cameroon [41] and *cyp6n1* has been reported to be overexpressed in *A. gambiae* after exposure to insecticides [42], it is tempting to speculate that these genes are rather involved in detoxification processes. However, many insect *cyp4g* have been associated with diverse functions different from detoxification of exogenous compounds. The closest gene of *cyp4g16* in *D. melanogaster*, *cyp4g15*, which is expressed in the larval brain [43] as well as *cyp4c15* in the crayfish *Orconectes limosus* [32] have been postulated to play a role in ecdysteroid metabolism rather than detoxification. Similarly, another gene, *cyp4g1* is highly expressed in the steroidogenic organ in *D. melanogaster* larvae and might be involved in lipid metabolism, which may indirectly regulate ecdysone biosynthesis [44]–[45]. *Cyp4g25* of the silkmoth *Antheraea yamamai* is expressed in the integument of larvae and seems to be in relation to diapause [46]. *Cyp4g16* and its closest homologs in insect species seem therefore to be linked to steroidogenesis, even if this enzyme is probably not a steroidogenic enzyme *stricto sensu*.

Transient knock-down of *cyp307a1* in BF mosquito females leads to a decrease of ovarian ecdysteroid production, further demonstrating that this gene is required for 20E biosynthesis in adult steroidogenic tissues. The involvement of *cyp307a1* in steroidogenesis in *A. gambiae* fits well with the previous identification of *cyp307a* genes being involved in steroid biosynthesis in *D. melanogaster*, *B. mori* and *Manduca sexta* and more recently in *Tribolium castaneum* [13], [18]–[19], [29], [31]. Up to now, the precise enzymatic activity of CYP307 proteins has not been elucidated. The evolutionary history of the *cyp307* family is quite complex and occurrence and expression pattern during development can vary depending on species [20], [31], [40], [47]. For instance, *Drosophila* carries two paralogs, *cyp307a1*, which is expressed only in embryos and in the follicle cells of ovary but not during the larval stages, and *cyp307a2*, which is expressed only during larval stages within the PG cells [19]. In contrast, in *Tribolium*, *cyp307a1* is expressed in embryos, larvae and adult females while *cyp307b1* is only expressed in the male accessory glands [31]. Like *Tribolium*, *A. gambiae* possesses *cyp307a1* and *cyp307b1* paralogs [20]. By RT-PCR, *cyp307a1* is detected in larvae, nymphs, adult females and males, but not in embryos, while *cyp307b1* is detected at every developmental stage (data not shown). In our microarray analysis, although *cyp307b1* was detected in ovaries and MRTs, its expression did not significantly vary in adult steroidogenic organs. The reason, if any, why evolution has allowed flexibility for *cyp307* genes is still not clear. The *cyp307* paralogs show the highest degree of identity between all steroidogenic CYP proteins and ectopic expression of *cyp307a1* rescues *Drosophila cyp307a2* mutants [19], [31], [47]. Although subtle catalytic differences may exist between CYP307 enzymes, these conserved paralogs are likely to be functionally redundant products of gene duplications that occupy different spatio-temporal patterns of expression to precisely control ecdysteroid titers during development [20], [40]. In *A. gambiae* adults, *cyp307a1* is highly up regulated in active steroidogenic tissues and this highlights that this gene must encode one of the early steps of steroidogenesis which are known to be more tightly regulated than the last steps leading to 20E [4]. This is consistent with results obtained in *D. melanogaster* indicating that they could act in the currently uncharacterized black box, from which one or multiple steps are believed to limit the production of ecdysone, in that no stable intermediate has been yet identified [4], [17]. No conversion of C or 7dC was observed in S2 cells transfected with *cyp307a1* but since the black box is supposed to contain several oxidative transformations, unless this gene catalyzes the initial reaction, expressing *cyp307a1* alone with 7dC would not be expected to produce a product [18]–[19], [48].

In conclusion, our approach has led to the identification of *cyp307a1* as playing a role in steroid biosynthesis in the malaria mosquito *A. gambiae*. Our study did not reveal any other CYP gene except *cyp307a1* that could be involved in this metabolic pathway, provided that the early steps are regulated at the transcriptional level. Although several studies strongly implicated *cyp307* genes in ecdysteroid biosynthesis and more particularly in the black box, additional experiments are necessary to clarify their precise biochemical activity. The recent identification of *Sro* as also playing a role in the black box [22] plus the availability of new ecdysteroid intermediates [17] should facilitate the characterization of these mysterious steps in the near future.

## Materials and Methods

### Mosquito strains

Two different *A. gambiae* strains were used for the microarray experiments: the *Kisumu* strain (molecular S form, from Kisumu, Western Kenya) and the *Yaoundé* strain (molecular M form, from Yaoundé, Cameroon). Based on population genomic evidences, it has been recently proposed to assign distinct species names to *A. gambiae* M and S forms [49]. The S form should conserve the *A. gambiae s.s*. name while the M form should now be *A. coluzzii*.

For all other experiments, only the *Yaoundé* strain was used. Mosquitoes were reared at 27°C under standardized conditions of 70% relative humidity and 12/12 h light/dark cycle, on 10% w/v sucrose solution.

### Microarray experimental design and sample collection

The microarray used in this study contains probes for 103 P450s, 31 COEs, 35 GSTs, 41 Red/Ox genes, 5 ATP-binding-cassette transporters, tissue-specific genes and housekeeping genes of the *Kisumu* strain of *A. gambiae* [33]. This array was used with different species of the *Anopheles* gambiae complex and exhibited similar performance between species [41]. Thus, we were confident that hybridizations with the *Yaoundé* strain of *A. gambiae* would be similar to the ones with the *Kisumu* strain. At most, the number of candidate genes in female experiments would be underestimated. To identify steroidogenic genes, we compared transcription levels of genes encoding CYP between steroidogenic tissues (ovaries from blood-fed females and male reproductive tracts, MRTs) and non steroidogenic tissues (ovaries from non blood-fed females). Each set of microarray experiment consisted of four hybridizations comprising two biological replicates (ovaries from BF females or MRTs) compared to a unique reference with dye swap of Cy3 and Cy5 fluorophores. The reference is a pool of ovaries from 3 independent cohorts of 3 days-old non-blood-fed females (n = 30 per cohort) either *Yaoundé* (female experiments) or *Kisumu* (MRTs experiments). For each biological replicate, about 300 adult mosquitoes synchronized at emergence were reared simultaneously. Each biological replicate consisted of mosquitoes (n = 30) from distinct generations to take into account stochastic variations.

For female experiments, ovaries from NBF females of the *Yaoundé* strain (reference in female experiments) were compared to ovaries of blood-fed (BF) females of the *Yaoundé* strain at 5 h, 16 h and 22 h post-blood-meal (PBM). 3 days-old females were allowed to feed on blood for 20 minutes. Partially or unfed females were discarded. For MRTs experiments, ovaries from NBF females of the *Kisumu* strain (reference in MRTs experiments) were compared to MRTs from 3 days-old males of the *Kisumu* strain. Ovaries from NBF females, non steroidogenic, were also used as the reference as MRTs have been shown to be steroidogenic during all the life of adult males [26]. Ovaries and MRTs were dissected in PBS (0.22 µm filtered) and stored in RNAlater (Applied Biosystems) at 4°C until RNA extraction.

### Target preparation and microarray hybridizations

RNA extractions, antisense RNA (aRNA) synthesis, and labelling reactions were performed independently for each replicate to take into account technical variation. Total RNA was extracted from batches of 40 to 60 ovaries/MRTs using the Picopure RNA isolation kit (Arcturus) with a DNAse treatment according to manufacturer's instructions. A batch contained mosquitoes from the same generation, collected on the same day. Total RNA quantity and quality were assessed by using a Nanodrop spectrophotometer (Nanodrop Technologies, Oxfordshire, U.K.) and agarose gel electrophoresis. From 1 (MRTs) to 5.5 µg (ovaries) of total RNA from each batch were amplified in one amplification round using the Riboamp RNA Amplification Kit (Arcturus) to generate purified aRNA. aRNA quantity and quality were further assessed by a Nanodrop spectrophotometrer and agarose gel electrophoresis. Final target preparation (aRNA fluorescent labelling and purification), hybridizations and microarray scanning were performed as previously described [50].

### Microarray data analysis

Data analysis was performed as described in David *et al.* [33] except that genes showing a *t* test P value<0.05 and an expression ratio >1.5-fold in either direction were considered differentially transcribed. In our screen, only genes overexpressed in steroidogenic tissues with an expression ratio >1.5-fold were further considered.

As a control, a calibration experiment was performed in which two aliquots of labelled aRNA derived from the same sample were co-hybridized to two arrays with dye-swap. As expected, none of the gene probes came out significantly differentially transcribed, supporting the statistical approach described above (data not shown).

All microarray data have been deposited at ArrayExpress (E-MTAB-1697).

### mRNA expression analysis by RT-PCR

4-day-old male tissues were carefully dissected in ice-cold, RNase-free phosphate buffered saline (100 mM, pH 7.4), containing 0.1% Tween (PBT). Total RNA was then extracted with SV Total RNA Isolation System (Promega) and quantified by spectrometry at 260 nm. cDNAs were generated using M-MLV reverse transcriptase from 100 ng of total RNA. *rpL17A*, coding for the ribosomal protein rpL17A, a domestic gene, was used as internal control.

For mRNA ovarian expression analysis after transient RNAi, ovaries from 10 BF females were subjected to RNA extraction. cDNAs were then generated from 500 ng of total RNA. Sense and antisense primers used for PCR analysis are located inside and outside the dsRNA sequence respectively, to avoid any amplification of dsRNA. *rpS7*, coding for the ribosomal protein S7, was used as internal control. Sequences of all primers used are given in Table S1.

### Gene cloning

Total RNA was isolated with Trizol reagent (Invitrogen) from vitellogenic ovaries and reverse transcribed with M-MLV reverse transcriptase (Promega). The *A. gambiae* genome is sequenced and genomic data are available on the website http://www.Ensembl.org/. Full length cDNA sequences of Ag*cyp314a1*, Ag*cyp307a1*, Ag*cyp6n1*, Ag*cyp4g16* were amplified from total cDNAs by PCR with specific primers (see Table S1). cDNAs were gel purified, cloned into pIB/V5-His (TA cloning, Invitrogen) and insert sequences verified (Genome Express; GenBank Accession numbers KF656700, KF656701, KF656702). E*gfp* (described in [51]) was cloned into pGEM-T Easy vector (pGEM-GFP) with specific primers (Table S1).

### *In situ* hybridization

MRTs were carefully dissected in PBT and fixed with 4% paraformaldehyde. RNA probes and *in situ* hybridization on MRTs from 4-day-old males were performed according to the method described in Parvy *et al.* [27]. Probes were synthesized from Ag*cyp307a1*, Ag*cyp6n1* and Ag*cyp4g16* full-length cDNA cloned into pGEMT-easy.

### Preparation of dsRNA, injection procedure, and sample preparation

cDNA fragments corresponding to the C-terminal sequence of *cyp307a1* (783 bp), *cyp314a1* (788 bp) and to E*gfp* were produced by RT-PCR using pIB/V5-*cyp307a1*, pIB/V5-*cyp314a1* and pGEM-GFP respectively as a template and gene-specific primers extended with a T7-promoter sequence containing a purine tail (Table S1). Those amplicons were then used as template to generate dsRNA by *in vitro* transcription (MEGAscript RNAi Kit, Ambion). dsRNA concentration and quality were estimated by spectrometry at 260 nm and electrophoresis on an ethidium bromide containing agarose gel. dsRNA were injected into one-day-old cold-anesthetized virgin females using a nanoject micro-injector (Drummond Scientific). 800 ng of dsRNA in 120 nl of water were injected per mosquito. On day 4 post-injection, injected virgin females were allowed to feed on mouse blood for 30 minutes; unfed females were discarded just after the blood meal. Ovaries from females were then carefully dissected 22 h after the blood meal and were subjected to RNA extraction/RT-PCR or *in vitro* incubation for ecdysteroid quantification. Experiments were performed on 2 independent cohorts of mosquitoes.

### *In vitro* culture and ecdysteroid quantification

Cultures were performed according to the method described in Pondeville *et al.* [26] except that ovaries were incubated for 3 h at 25°C. After incubation, culture medium was collected and stored at −20°C until ecdysteroid quantification.

Ecdysteroids were quantified by EIA, with 20-hydroxyecdysone-2-succinate coupled to peroxidase as a tracer (dilution 1∶80,000) and either the L2 antiserum (a generous gift from Dr. M. De Reggi, dilution 1∶40,000) or the EC19 antiserum (a generous gift from Dr. J.-P. Delbecque, dilution 1∶10,000). The L2 antibody recognizes both E and 20E, as calculated from the comparison of reference standard curves (data not shown). The EC19 antibody recognizes only 20E. Calibration curves were generated with E or 20E (3.6 to 500 pg/tube) diluted in Schneider's medium and the *in vitro* production was expressed in E or 20E equivalents. Under these conditions, detection limits are 7 pg E equivalents for the L2 antibody and 5 pg 20E equivalents for the EC19 antibody. Ecdysteroids secreted by tissues were measured directly on incubation media. For each sample, measurements were performed in duplicate and the results are expressed as mean values ± S.E.M. of several (n = 20) independent ovary pairs. All experiments have been repeated on 2 independent cohorts of mosquitoes. Data were subjected to statistical analysis using Mann-Whitney test.

## Supporting Information

Table S1 **Primers used in the study.**
(XLS)
